# The Science and Art of Surgery

**Published:** 1854-07

**Authors:** 


For sale by MILLER, ORTON & MULLIGAN. H.

ART. IX.—*The Science and Art of Surgery. Being a Treatise on Surgical Injuries, and Operations.* By JOHN ERICHSEN, Prof. of Surgery in University College and Surgeon to University College Hospital. Edited by JOHN H. BRINTON, M. D. Philadelphia: Blanchard & Lea. 1854.

The fly-leaf facing the title page has upon it this pithy aphorism from Lord Bacon on Learning: "They be the best Chirurgeons which being learned incline to the traditions of experience, or being empirics incline to the methods of learning." We leave this sentence to the thoughtful consideration of our readers. It is too suggestive to need comment.

The author is the successor of Liston in the wards of the University College Hospital. He has now for many years occupied this distinguished position, and is, of course, able to quote a very large experience in surgical science and art. He has here given us a volume of some nine hundred pages, accompanied by many, and very clever, wood-cuts. Some subjects, such as diseases of the eye and skin, are omitted, as being special subjects requiring too much space for this work.

We are disposed to receive favorably this new candidate for surgical fame. In the cursory examination we have given it, we have been pleased with its arrangement and style. The doctrines of Paget prevail in matters of surgical pathology, and this is, to us, an additional reason for liking the work. Our readers will find it superior to many of the popular works, and we are inclined to believe that it will have a prosperous circulation, even in the present crowded condition of surgical literature.

